# International Physical Activity Questionnaire (IPAQ) and New Zealand Physical Activity Questionnaire (NZPAQ): A doubly labelled water validation

**DOI:** 10.1186/1479-5868-4-62

**Published:** 2007-12-03

**Authors:** Ralph Maddison, Cliona Ni Mhurchu, Yannan Jiang, Stephen Vander Hoorn, Anthony Rodgers, Carlene MM Lawes, Elaine Rush

**Affiliations:** 1 Clinical Trials Research Unit, University of Auckland, Auckland, New Zealand; 2 Centre for Physical Activity and Nutrition, Auckland University of Technology, Auckland, New Zealand

## Abstract

**Background:**

Accurate measurement of physical activity is a pre-requisite for monitoring population health and for evaluating effective interventions. The International Physical Activity Questionnaire (IPAQ) is used as a comparable and standardised self-report measure of habitual physical activity of populations from different countries and socio-cultural contexts. The IPAQ has been modified to produce a New Zealand physical activity questionnaire (NZPAQ). The aim of this study was to validate the IPAQ and NZPAQ against doubly labelled water (DLW). Method: Total energy expenditure (TEE) was measured over a 15-day period using DLW. Activity-related energy expenditure (AEE) was estimated by subtracting the energy expenditure from resting metabolic rate and thermic effect of feeding from TEE. The IPAQ (long form) and NZPAQ (short form) were completed at the end of each 7-day period. Activity-related energy expenditure (IPAQ_AEE _and NZPAQ_AEE_) was calculated from each questionnaire and compared to DLW_AEE_.

**Results:**

Thirty six adults aged 18 to 56 years (56% female) completed all measurements. Compared to DLW_AEE_, IPAQ_AEE _and NZPAQ_AEE _on average underestimated energy expenditure by 27% and 59%, respectively. There was good agreement between DLW_AEE _and both IPAQ_AEE _and NZPAQ_AEE _at lower levels of physical activity. However there was marked underestimation of questionnaire-derived energy expenditure at higher levels of activity.

**Conclusion:**

Both the IPAQ and NZPAQ instruments have a demonstrated systematic bias toward underestimation of physical activity-related energy expenditure at higher levels of physical activity compared to DLW. Appropriate calibration factors could be used to correct for measurement error in physical activity questionnaires and hence improve estimation of AEE.

## Background

The global estimate for physical inactivity (doing very little or no physical activity at work, at home, for transport or in discretionary time) is 17% [1], whereas the estimate for insufficient levels of physical activity (< 150 minutes moderate or < 60 minutes of vigorous activity per week) is 40% [1]. Lack of physical activity is associated with an increased risk of ischemic heart disease, type 2 diabetes, colon cancer, depression, and breast cancer [1]. Accurate measurement of physical activity is a pre-requisite for monitoring population health and evaluating effective interventions, with the preferred and most practical method in epidemiological studies being self-report questionnaires.

The International Physical Activity Questionnaire (IPAQ) is used as a standardised measure to estimate habitual practice of physical activities of populations from different countries and socio-cultural contexts [2,3]. Two-forms of the IPAQ have been developed: a short and a long version, both of which involve 7-day recall of physical activity. The short-form (SF) was designed for use in surveillance studies, in which time is limited, and consists of 8 items to estimate the time spent performing physical activities (moderate to vigorous) and inactivity (time spent sitting). The long form (LF) was designed to provide a comprehensive evaluation of daily physical activities, and assesses the time spent walking, doing moderate-intensity and vigorous-intensity activity within the domains of work, transportation, domestic and gardening (yard) activities, and leisure-related activities.

In 2003, Craig et al [3] provided reliability and validity data from 12 countries for both the long-and short-forms, showing that the IPAQ questionnaires produced reliable data, as well as acceptable criterion validity against the CSA accelerometer (rs approximately 0.30). Although a number of reliability and validity studies have been conducted [3-7], the IPAQ has not yet been validated against the gold (criterion) standard of doubly labelled water (DLW) [8]. The DLW technique is a biochemical procedure used to assess total energy expenditure (TEE) through biological markers that reflect the rate of metabolism in the body [9]. Combined with the measurement of resting metabolic rate (RMR) this technique is considered the criterion measure of physical activity-related energy expenditure.

The New Zealand (NZ) Ministry of Health and Sport and Recreation New Zealand (SPARC) recently developed two self-report questionnaires, The NZ Physical Activity Questionnaire (NZPAQ): both a more in-depth long form (LF) and a briefer short-form (SF). The NZPAQ-SF was developed as a surveillance tool and is a modified version of the IPAQ-SF. The modifications were intended to increase the relevance and cultural appropriateness of the instrument for New Zealand. The NZPAQ-SF questions assess the three dimensions of physical activity, 'frequency, duration, and intensity'. The format for the first 7 questions is for participants to recall the frequency they performed brisk walking, moderate, vigorous and a combination of both over the past 7 days. If indicated, participants then record the duration (time) they performed the activity. Question 8 is optional and indicates stage of exercise behaviour change. Visual show-cards are provided to the participant to given an indication of moderate and vigorous activities. The most important differences between the instruments are (1) the IPAQ-SF does not measure the frequency of activity, by contrast, the NZPAQ-SF includes an extra item to tap this dimension of physical activity. (2) The order of items in the NZPAQ-SF have been reversed compared to the IPAQ-SF. (3) The NZPAQ-SF does not include an item on sedentary behaviour and focuses on physical activity only.

In a validation study, total physical activity (including walking and moderate, and vigorous intensity activities) measured using the NZPAQ-SF was correlated with that measured using the IPAQ-LF (r = 0.33 p < 0.0001). However physical activity levels were overestimated at low activity levels and underestimated at high activity levels when compared to heart-rate derived measurements [10]. The indication for use of the IPAQ-LF is for research whereas the IPAQ-SF is for surveillance purposes. Because of the similarity between the IPAQ-SF and NZPAQ-SF we chose the latter for this study. The aim of this study was to validate both the IPAQ-LF and NZPAQ-SF against DLW.

## Methods

### Study participants and recruitment

Adults living in Auckland, New Zealand were eligible if they were aged between 18 and 65 years, able to give written informed consent, able to communicate in English, and weight stable (not dieting or had not experienced recent dramatic changes in weight). Potential participants were recruited via community advertisements, direct contact through the university, and word of mouth. Adults who used a walking aid (crutches, cane, or wheelchair) or who were unable to perform physical activity for medical reasons were excluded. The study protocol and related documents were approved by the Northern Regional Ethics Committee (NTY/06/02/011).

### Overview of procedures

Each participant was assessed over a 15-day period. At baseline (Day 0), measurements were taken of height, weight, and resting metabolic rate (RMR). Baseline urine samples were collected prior to administration of DLW. Participants returned at days 1, 8, and 15 for spot urine sample collection and questionnaire completion.

### Measures

#### Doubly labelled water (DLW)

DLW is considered the reference or gold standard for the assessment of total energy expenditure (TEE). When combined with a measure of resting metabolic rate (RMR), activity-related energy expenditure (AEE) can be calculated [11]. In this study TEE was determined using the DLW method [12,13]. After a baseline urine sample was collected for determination of background isotope enrichment (Day 0), each participant ingested a single dose of DLW (^2^H and ^18^O; Cambridge Isotope Laboratories, Inc., Andover, MA), corresponding to 0.12 g of 99.9% of ^2^H_2_O and 2 g of 10% ^18^O per kg body weight. The dose was followed by the ingestion of two 50-mL washes of the container with tap water. Post enrichment urine samples were collected the following day (Day 1) and at Days 8 and 15. A trained research assistant collected all specimens and recorded the exact time of voiding. Samples were frozen at -21°C until assayed for ^2^H and ^18^O. A subsample of the dose was reserved and after appropriate dilution analyzed at the same time as the urine samples to serve as a reference point. Samples were analysed in triplicate using a TC/EA (Thermo Chemical Elemental Analyser) interfaced (ConFlo II; Thermo Finnigan, Bremen, Germany) to an isotope ratio mass spectrometer (Delta Plus XP; Thermo Finnigan, Germany). TEE was calculated using the two point approach with an assumed respiratory quotient (RQ) of 0.85 over the 14 d period [14]. Activity-related energy expenditure (AEE) was calculated as the difference between TEE and RMR. For the purpose of this study, diet-induced thermogenesis (DIT) was considered to be 10% of TEE [11].

#### Body composition

Anthropometric measurements were taken in the morning of Day 1. Height was measured to the nearest 0.1 cm with a portable stadiometer (Harpenden Stadiometer, Chasmors Ltd, London) and weight was measured to the nearest 0.1 kg on an electronic scale (Salter Scales). To assess body composition, resistance, reactance, impedance and phase were determined using single-frequency (50 kHz) bioimpedance analysis (Model BIM4, Impedimed, Capalaba, Australia) using a standardized protocol. The average of three repeated measurements of resistance agreeing to within 1 ohm of each other was used in subsequent analyses. Lukaski [15] equations were used to determine fat free mass (FFM) for European participants and Swinburn [16] equations to determine fat mass for Maori and Pacific Island participants. Fat mass (FM) was derived as the difference between body weight and FFM. Percentage of body fat was calculated as FM/body weight × 100.

#### Resting Metabolic Rate (RMR)

RMR was measured by indirect calorimetry (Day 0) using the MetaMax^®^3B (Cortex, Biophysik) portable gas exchange analysis system, a device with demonstrated precision [17,18]. This methodology incorporates the fraction of inspired oxygen and expired O_2 _and CO_2_, pump rate, ambient conditions in association with the Weir equation [19] to calculate RMR. Participants were instructed to limit any vigorous physical activity the day before RMR measurement, and tests were conducted in the morning after an overnight fast. A face mask (Hans Rudolf, Kansas City, MO) held in place by a nylon harness covered the participant's nose and mouth. The mask was attached to a bidirectional digital turbine flow meter to measure the volume of inspired and expired air. A sample line between the turbine and analyzer unit determined O_2 _and CO_2 _content. A two point calibration procedure was conducted before each testing session according to the manufacturer's guidelines (Cortex Biophysik MetaMax^®^3B Manual). Participants rested for a period of 20 minutes in the supine position before RMR testing, during which breath by breath measurements were recorded for 30 minutes. A coefficient of variation of less than 10% of consecutive energy expenditure measurements was used as steady state [9].

#### Physical activity questionnaires

The IPAQ-LF was administered at day 0, day 8, and 15. The questionnaire took approximately 15 minutes to complete, and a trained research assistant was present to assist participants with the questionnaire.

The NZPAQ-SF was administered at day 8 and 15. Participants indicated the number of times in the past seven days they spent walking, and in moderate, and vigorous activity) and also the usual amount of time spent per day. Physical activity show cards provided examples of the intensity of various physical activities (e.g., Rugby Union/carrying heavy loads = vigorous, doubles tennis/lawn mowing = moderate). NZ Maori (indigenous New Zealanders)-specific activities are included in this questionnaire. The questionnaire took approximately 10 minutes to complete and a trained research assistant was present to assist participants.

#### Energy expenditure estimation

Physical activity data obtained from the IPAQ-LF and NZPAQ-SF were computed for metabolic equivalent (MET)-minutes per week, calculated as the MET intensity multiplied by the minutes for each activity over the seven day period. METs are multiples of the RMR and a MET-minute is computed by multiplying the MET score of an activity by the minutes. It was assumed that 1 MET was equivalent to 1 kcal/kg/hr for all participants [20]. Adopting this approach, the MET values (e.g. 3.3) were multiplied by the participant's body weight (kg) and then divided by 60 minutes to convert the unit into kcal/min (e.g. 3.3 * 50 kg/60 min). Energy expenditure values were then converted to kJ (1 kcal = 4.18 kJ) for direct comparison with DLW. Energy expenditure values from week 1 (Days 1–7) and week 2 (Days 8–15) for the IPAQ-LF and NZPAQ-SF, respectively were summed and divided by the number of days to provide average daily physical-activity related energy expenditure.

#### Statistics

Statistical analysis was performed using SAS (Statistical Analysis Systems) version 9.1.3 and S-PLUS 6.1 for Windows. Means and standard deviation (SD) were calculated. The significance level was set at 5%. Average daily activity-related energy expenditure (AEE) measured using DLW, IPAQ-LF, and NZPAQ-SF was calculated for each participant. Bland-Altman plots were first used to evaluate the level of agreement in activity-related energy expenditure (kJ) between DLW and the different measures. Spearman correlation coefficients were then calculated to assess the degree of linear association between measurements. Regression analysis was finally conducted to explore the relationships between the dependent variable (DLW derived AEE) and independent variable (AEE derived from the IPAQ-LF and NZPAQ-SF, respectively). Potential covariates of interest (age, sex, weight, fat free mass) were considered and kept in the regression model if they were statistically significant.

To examine the level of agreement between the IPAQ-LF, NZPAQ-SF, and DLW we produced Bland-Altman plots [21,22]. For each series, the Y axis represents the difference in AEE between each measure (IPAQ and NZPAQ) and DLW. The *x *axis of these plots represents the AEE from DLW. The limits of agreement (dotted lines) equalling two standard deviations of the mean difference above and below the mean (solid line) were plotted.

## Results

Participants were 36 adults (20 female and 16 males) aged 18 to 64 years (Table 1). The participants were, on average 39 years old, predominantly of European ethnicity (75%). According to international [23] BMI cut-off points, participants were on average overweight (Mean BMI = 25.9 kg/m^2^), which is typical of the New Zealand population (BMI = 26.1 kg/m^2^) [24]. No sex differences were found for BMI, percent body fat, weight, TEE, AEE. The males, however, had significantly more lean body mass (62 kg) than the females (48 kg) (p < 0.001).

**Table 1:** Participant's characteristics

Demographics	Mean	SD	Min	Max
Age (yr)	39	10	18	64
Height (cm)	171.5	7.4	159.0	185.0
Weight (kg)	75.9	14.8	55.2	122.0
BMI (kg/m2)	25.7	4.0	19.9	37.2
FM (kg)	21.20	8.30	6.31	38.67
FFM (kg)	54.74	10.30	40.66	85.54
Sex (n/%)				
Female	20 (56%)			
Male	16 (44%)			
Ethnicity (n/%)				
NZ European	27 (75%)			
NZ Maori	4 (11%)			
Tongan	1 (3%)			
Asian	2 (6%)			
Other	5 (15%)			

Reliability of the measures (IPAQ and NZPAQ) The test-retest reliability coefficients of the IPAQ-LF questionnaires were acceptable with Spearman correlation coefficients of 0.79 (p < 0.0001) between 0 and 8 days and 0.74 (p < 0.0001) between days 8 and 15. A similar correlation coefficient was found for the NZPAQ-SF questionnaire between days 8 and 15 (0.69, p < 0.0001). Both parametric and non-parametric tests were conducted and revealed no significant differences in MET-minutes per week from baseline to Day 8 for the IPAQ, suggesting physical activity behaviour was not affected by participation in this study

### Energy expenditure

**Figure 1:**
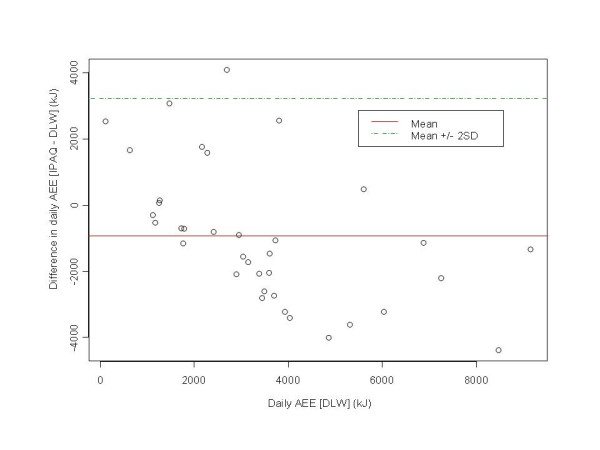
Difference between AEE determined with DLW and IPAQ-LF.

**Figure 2:**
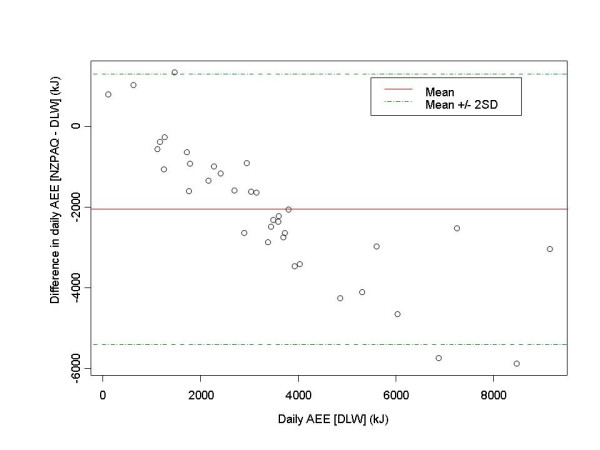
Difference between AEE determined with DLW and NZPAQ-SF.

AEE values for DLW, IPAQ-LF, and NZPAQ-SF are presented in Table 2. The correlations between IPAQ_AEE_, NZPAQ_AEE_, and DLW_AEE _were of a moderate magnitude (rs = 0.31–0.38, respectively). Because AEE values (kJs) were derived from MET/min estimates from the IPAQ-LF and NZPAQ-SF correlations were high (Table 3). At the group level, compared to DLW_AEE_, mean IPAQ_AEE _and NZPAQ_AEE _underestimated energy expenditure by 27% and 59%, respectively. At the individual, compared to DLW_AEE _there was overestimation of energy expenditure with the IPAQ-LF for 7 participants at lower levels of physical activity (equivalent to 376 daily MET/min per day). However for the remaining participants, as activity levels increased the IPAQ-LF underestimated AEE compared to DLW_AEE _(Figure 1) from approximately 1000 kJ (equivalent to 187 MET/min per day). Results were similar for NZPAQ_AEE _with marked underestimation of energy expenditure as levels of activity increased from approximately 1000 kJ (Figure 2).

**Table 2:** Energy expenditure (Means ± SD)

	DLW		IPAQ-LF	NZPAQ-SF	IPAQ-LF	NZPAQ-SF
	TEE kJ	AEE kJ	AEE kJ	AEE kJ	MET/min	MET/min
Females (n= 20)	12174 ± 2695	3628 ± 1957	2225 ± 1892	1318 ± 1040	439 ± 336	269 ± 206
Males (n = 16)	13603 ± 3112	3224 ± 2377	2855 ± 2224	1497 ± 1372	503 ± 397	261 ± 232
Total (n = 36)	12809 ± 2935	3448 ± 2131	2505 ± 2041	1398 ± 1184	467 ± 361	265 ± 214

Note:

DLW = Doubly labelled water

LF = Long form

SF = Short form

TEE = Average total energy expenditure per day

AEE = Average activity-related energy expenditure per day

MET/Min = Daily Metabolic Equivalent Minutes

**Table 3:** Spearman Correlation Coefficients between Different EE Measures

	DLW_AEE_	IPAQ_AEE_	NZPAQ_AEE_	IPAQ Met/Min	NZPAQ Met/Min
DLW_AEE_	1.00	0.31*	0.38**	0.33**	0.39**
IPAQ_AEE_		1.00	0.74***	0.98***	0.66***
NZPAQ_AEE_			1.00	0.71***	0.96***
IPAQ Met/Min				1.00	0.69***
NZPAQ Met/Min					1.00

* p = 0.06,

** p < 0.05

*** p < 0.01

Due to the demonstrated systematic bias toward underestimation of AEE at higher levels of physical activity, regression analyses were conducted where DLW_AEE _was the dependent variable and the respective self-report measures were each used as independent variables (Table 4). In addition, because the NZPAQ-SF was developed from the IPAQ we conducted a regression analysis with IPAQ_AEE _as the dependant variable and NZPAQ_AEE _as the independent variable (Table 4, Set 3). Potential covariates of interest (age, sex, weight, fat [kg], and FFM) were considered for an adjusted analysis. Except for sex and FFM (IPAQ vs. NZPAQ), all other covariates were highly non-significant and therefore dropped from the regression model. Approximately 38% of the variance in DLW_AEE _was explained by the IPAQ-LF and NZPAQ-SF, respectively. The NZPAQ_AEE _explained approximately 53% of the variance of the IPAQ_AEE_.

**Table 4:** Analysis of Covariance (Regression Equation)

Set 1			
Dependent Variable (Y):	DLW average daily activity-related EE (kJ)		
Independent Variable (X):	IPAQ average daily activity-related EE (kJ)		
Parameter	Estimate	StdErr	*p*-value (*t*-test)
Intercept	2137.01	497.95	0.000
IPAQ_AEE_	0.52	0.16	0.002
R^2 ^= 0.25			
Regression Line: DLW_AEE _= 2137 + 0.52 * IPAQ			
With covariates			
Intercept	-2082.47	2009.59	0.31
IPAQ_AEE_	0.45	0.15	0.006
Male	-2589	830.44	0.017
FFM	97.37	42.24	0.028
*R*^2 ^= 0.38			
Regression Line: DLW_AEE _= -2082 + 0.45 * IPAQ_AEE _+ 2589 * sex + 97 * FFM			

Set 2			
Dependent Variable (Y):	DLW average daily activity-related EE (kJ)		
Independent Variable (X):	NZPAQ-SF average daily activity-related EE (kJ)		

Parameter	Estimate	StdErr	*p*-value (*t*-test)
Intercept	1885.39	440.40	0.0001
NZPAQ_AEE_	1.18	0.24	0.0001
*R*^2 ^= 0.38			
Regression Line: DLW = 1885 + 1.18 * NZPAQ_AEE_			

Set 3			
Dependent Variable (Y):	IPAQ-LF average daily activity-related EE (kJ)		
Independent Variable (X):	NZPAQ-SF average daily activity-related EE (kJ)		

Parameter	Estimate	StdErr	*p*-value (*t*-test)
Intercept	848.32	360.67	0.037
NZPAQ_AEE_	1.18	0.22	0.0001
*R*^2 ^= 0.47			
Regression Line: IPAQ = 848 + 1.18 * NZPAQ			

With covariates			
Intercept	-1135.67	1080.36	0.30
NZPAQ_AEE_	1.25	0.21	0.0001
Age	49.09	25.06	0.05
*R*^2 ^= 0.53			
Regression Line: IPAQ_AEE _= -1135 + 1.25 * NZPAQ_AEE _+ 49 * Age			

## Discussion

To the best of our knowledge, this is the only study to date to provide DLW validation of the IPAQ-LF and NZPAQ-SF. Compared with DLW, our data showed that both self-report measures on average underestimated physical activity-related energy expenditure especially at higher levels of physical activity. Although accurate measurement of daily free-living physical activity is important to describe the behaviour, its impact on health, and to assess the effectiveness of interventions, only a handful of studies have tested the ability of different epidemiological methods to assess EE in free-living individuals [25,26].

Compared to DLW_AEE_, on average, the IPAQ-LF underestimated activity-related energy expenditure. There was good approximation at the lowest levels of physical activity (1000–2000 kJ or equivalent to ~187–376 daily MET/mins), with greater underestimation as physical activity increased (greater than 3000 kJs or ~565 daily MET/mins). Similar findings were found for the NZPAQ-SF, except that there was a systematic underestimation starting at 1000 kJ, with greatest underestimation at over 5000 kJ (~942 daily MET/min). The best overall approximation of AEE with DLW was with the IPAQ-LF with a 27% deficit, compared to the 59% deficit of the NZPAQ-SF. These findings are comparable with other studies that have shown that 7-day recall questionnaires (e.g., Cross-Cultural Activity Participation Study Typical Week Activity Recall questionnaire; Minnesota Leisure Time PA questionnaire; College Alumni Score) underestimated energy expenditure by 10–30% compared to DLW measurement [25-27]. However, other studies have shown that 7-day physical activity recall questionnaires (e.g., Stanford Seven Day Recall Questionnaire) overestimate energy expenditure compared to DLW in free-living adults [25,27-29].

The systematic bias in estimation of energy expenditure using the NZPAQ-SF is consistent with the NZPAQ development validation work, which showed self-reported physical activity levels were overestimated at low activity levels and underestimated at high activity levels in comparison to heart-rate derived measurements [10]. This bias may be due to the limited number and the nature of the questions in the short form, which asks participants to rate time spent in moderate to vigorous activities only. Light-intensity activities are not captured by items in the NZPAQ-SF but would be included in the DLW estimate. It is possible that various activities are not accurately recalled, either because people forget, or they cannot recall unstructured bouts of activity (e.g., such as climbing stairs, household work), thereby underestimating total physical activity.

Both physical activity questionnaires (IPAQ-LF and NZPAQ-SF) were not specifically designed to measure activity-related energy expenditure, rather MET values have been provided with the IPAQ scoring protocol to give an indication of levels of physical activity. In the present study we estimated AEE from MET minutes, which may have contributed to the underestimation of energy expenditure. For example, allocated MET values from the IPAQ scoring protocol may not reflect the intensity of all activities for all people. For example, an individual who indicates they walk 5 days a week for approximately 30 minutes/day equates to 150 minutes of activity. According to the IPAQ scoring protocol, MET-minutes/week = 3.3 × walking minutes × walking days in the week, which is equivalent to 495 MET-minutes/week. However one's walking speed and intensity vary according to many variables (e.g., weather, geography etc), and may in fact be greater or less than 3.3 METS [30], resulting in a misrepresentation of the energy cost of the activity. Future studies might choose to control for individual levels of physical fitness and exercise levels. Individuals with greater physical fitness, and who participate in large amounts of activity may perceive moderate and vigorous activity differently compared to those who are more sedentary, thereby confounding self-report-derived energy expenditure.

Although our results generally suggest the IPAQ-LF on average underestimated physical activity-related energy expenditure, simple adjustments to the energy expenditure estimations could be used to correct for this systematic bias. There was evidence supporting a linear relationship between AEE and IPAQ/NZPAQ, therefore, a crude approach to adjust energy expenditure would be to take the IPAQ estimate of AEE, divide by 2 and add approximately 2000 kJ. This approach does however suggest a constant difference in energy expenditure. Our regression analysis showed that including measures of fat free mass and sex improved the prediction of DLW_AEE _(explaining approximately 38% variance) using the IPAQ-LF, therefore more sophisticated adjustment approaches may need to consider these variables.

The use of correction factors for self-report measures of physical activity has implications for future research, particularly with the frequent use of these measures. Although a variety of methods are employed to assess physical activity behaviour, such as accelerometers and heart rate monitors, their use is often cost-prohibitive for epidemiological studies or public health surveillance. Both objective measures also have been shown to over-and under-estimate energy expenditure compared to DLW [28,31]. Thus the preferred method for population-level purposes in adults is self-reported instruments [32].

Self-reported physical activity is subject to reporting bias and as this study has shown, physical activity questionnaires do not accurately quantify activity-related energy expenditure. The development of an appropriate correction factor, which could be applied to questionnaires such as the IPAQ would overcome systematic bias and provide a more reliable and valid measure of energy expenditure estimation. At the population level the IPAQ has potential to standardise physical activity measurement. Used in conjunction with an appropriate correction factor the IPAQ would be valuable for (1) international comparison and (2) estimating the burden of disease associated with physical inactivity.

Previous research findings have shown that physical activity recall questionnaires both under-and over-estimate energy expenditure, however physical activity records may be more accurate [25]. The inherent difficulties associated with recalling physical activities may limit those data to estimate the energy expenditure associated with free living physical activity. Rather, 7-day recall questionnaires may be best suited to national and international level surveillance by providing information on the nature (type and intensity) and duration (time) of physical activity.

Similar to other validations studies using the DLW approach this study was limited by the small sample size (n = 36). Both the IPAQ and NZPAQ were validated in healthy, free-living adults and therefore the findings cannot be generalised to other populations. Strengths however, include the prospective study design and the use of DLW and RMR to assess AEE. This is also the first study to conduct DLW validation of the IPAQ.

In conclusion, the findings of this DLW validation study support the use of the IPAQ and NZPAQ as measures of physical activity for the purpose of epidemiological research, such as periodic national surveys. Overall, both instruments have a demonstrated systematic bias toward underestimation of physical activity-related energy expenditure (particularly the NZPAQ) at higher levels of physical activity. Appropriate calibration factors could be used to correct for this measurement error and hence improve estimation of AEE. No single valid, reliable field measure of physical activity exists which is logistically feasible for use in all population settings. Therefore, an understanding of the strengths and limitations of each technique is required before choosing the appropriate assessment method for a specific research question. Although recall questionnaires are the most practical means of measuring PA in large population studies, they both over-and under-estimate physical activity-related EE and, if using them for this purpose, calibration factors should be considered to correct for this.

## Authors' contributions

All authors have made a significant contribution to this manuscript. RM conducted the study, collected the data and prepared the manuscript. CNM, CL, and AR all contributed to the study design and revisions of the draft manuscripts. YJ and SV conducted the statistical analysis and reviewed draft versions of the manuscript. ER provided advice for the doubly labeled water, the body composition procedures and reviewed draft versions of the manuscript. All authors reads and approved the final manuscript.
